# Supplementary material for: Checkpoint Defects Elicit a WRNIP1-Mediated Response to Counteract R-Loop-Associated Genomic Instability
Source: Cancers (Basel). 2020 Feb 7;12(2):389. doi: 10.3390/cancers12020389 (PMC7072626; doi:10.3390/cancers12020389)
Supplement: Supplementary file 1 [file cancers-12-00389-s001.zip › Cancers SUPPL. MATERIALS AND METHODS.pdf]

CHECKPOINT DEFECTS ELICIT A WRNIP1-MEDIATED RESPONSE TO COUNTERACT R-LOOP-ASSOCIATED GENOMIC INSTABILITY

Veronica Marabitti[1], Giorgia Lillo[1], Eva Malacaria[1], Valentina Palermo[1], Pietro Pichierri[1] and Annapaola Franchitto[1, *]

[1]Department of Environment and Health, Section of Mechanisms Biomarkers and Models, Istituto Superiore di Sanita', Viale Regina Elena 299, Rome, 00161, Italy

Supplementary Materials and Methods

Cell cultures

HEK293T cells were obtained from American Type Culture Collection (VA, USA). HEK293T cells proficient and deficient for WRN were generated as previously described [1]. Cells were maintained in Dulbecco's modified Eagle's medium (DMEM; Life Technologies) supplemented with 10% FBS (Boehringer Mannheim), incubated at 37°C in a humidified 5% CO_2 atmosphere, and cultured in the presence of hygromycin (200 μg/ml) (EMD Chemicals Inc.) to maintain selective pressure for shRNA expression.

Chemicals

Chemicals used were commercially obtained for the replication stress-inducing drug: aphidicolin (Sigma-Aldrich), ATM inhibitor (KU-55933; Selleckchem), CHK1 inhibitor (UCN-01; Alexis Biochemicals) and the transcription elongation inhibitor 5,6-dichloro-1-ß-D-ribofurosylbenzimidazole (DRB, Sigma-Aldrich). The final concentrations of the drugs used were: 0.4 µM aphidicolin (Aph), 10 µM KU-55933, 300 nM UCN-01 and 50 µM DRB. Stock solutions for all of the pharmacological inhibitors were prepared in DMSO at a concentration of >1000-fold. The final concentration of DMSO in the culture medium was always < 0.1%.

Plasmids and RNA interference

The phospho-mimic (Flag-$CHK1^{317D/345D}$) mutant form of CHK1, a kind gift from Prof. K.K. Kanna (Queensland Institute of Medical Research, Australia), was constructed as previously described [2]. The construct used to perform RNaseH1 overexpression experiments is a generous gift from Prof. R.J. Crouch (National Institutes of Health, Bethesda, USA). As previously described [3], the GFP-tagged RNaseH1 plasmid was generated by introducing a mutation on Met27 abrogating mitochondrial localization signal (RNaseH1-M27). To express the plasmids, cells were transfected using the Neon™ Transfection System Kit (Invitrogen), according to the manufacturer's instructions.

WRN, WRNIP1, TopBP1, Claspin and XPG genetic knockdown experiments were performed by Interferin (Polyplus), according to the manufacturer's instructions. siRNAs were used at 15 nM. As a control, a siRNA duplex directed against GFP was used. Protein depletion was achieved using siRNAs (QIAGEN) targeting the 3'UTR regions of proteins: WRN (5'-CGGATTGTATACGTAACTCCA-3'); WRNIP1 (5'-ATGAATTAATGTTATAAGGAA-3'); TopBP1 (5'-CUCACCUUAUUGCAGGAGAdTdT-3'); Claspin (5'-

CCUUGCUUAGAGCUGAGUCdTdT-3'); XPG (5'- GAACGCACCUGCUGCUGUA-3'); Depletion of the proteins was confirmed by Western blotting using the relevant antibodies.

Co-immunoprecipitation and Western blotting analysis

Immunoprecipitation was performed as previously described [1]. Briefly, for co-immunoprecipitation (co-IP) experiments, exponential growing HEK293T cells were cultured overnight at a density of 2.5×10^6 per 150 mm Petri dish and treated or not as indicated. After treatment, cells were collected and centrifuged. The cell pellets were resuspended in lysis co-IP buffer (1% Triton X-100, 0.5% Na-dehoxycolate, 150 mM NaCl, 1 mM EGTA, 20 mM Tris/HCl pH 8.0), freshly supplemented with protease inhibitor cocktail (Thermo Scientific) and sonicated on ice. After centrifugation, for each IP sample, lysate was incubated with 20 µl anti-FLAG M2 magnetic beads (Sigma-Aldrich) at 4°C overnight. The IP reaction was washed three times with the co-IP buffer, incubated in 2× sample loading buffer (100 mM Tris/HCl pH 6.8, 100 mM DTT, 4% SDS, 0.2% bromophenol blue and 20% glycerol) for 30 min at 90°C then subjected to Western blotting. Cell lysates for Western blotting were prepared as previously reported [4]. Antibodies used for Western blotting were commercially obtained for WRN (Santa Cruz Biotechnology, 1:1000), WRNIP1 (Novus Biologicals, 1:2500), LAMIN B1 (Abcam, 1:40.000), Tubulin (Sigma-Aldrich, 1:10000), Claspin (Cell Signaling Technologies, 1:1000), TopBP1 (Bethyl, 1:1000), RAD51 (Abcam, 1:10000), GFP (Santa Cruz Biotechnology, 1:1000), phospho-CHK1-Ser345 (Cell Signaling Technologies, 1:1000), CHK1 (Cell Signalling Technologies, 1:1000), XPG (Santa Cruz Biotechnology, 1:1000), pATMS1981 (Millipore, 1:300), ATM (Novus Biologicals, 1:1000), Flag M2 (Sigma-Aldrich, 1:1000) and GAPDH (Millipore). Horseradish peroxidase-conjugated goat specie-specific secondary antibodies (Santa Cruz Biotechnology, Inc.) were used. Quantification was performed on scanned images of blots using ImageJ software, and values are shown on the graphs as indicated.

Alkaline Comet assay

DNA breakage induction was examined by alkaline Comet assay (single-cell gel electrophoresis) in denaturing conditions as described [5]. Cell DNA was stained with a fluorescent dye GelRed (Biotium) and examined at 40× magnification with an Olympus fluorescence microscope. Slides were analysed by a computerized image analysis system (Comet IV, Perceptive UK). To assess the amount of DNA damage, computer-generated tail moment values (tail length × fraction of total DNA in the tail) were used. A minimum of 200 cells was analysed for each experimental point. Apoptotic cells (smaller comet head and extremely larger comet tail) were excluded from the analysis to avoid artificial enhancement of the tail moment.

Chromosomal aberrations

Cells for metaphase preparations were collected according to standard procedure and as previously reported [6]. Cell suspension was dropped onto cold, wet slides to make chromosome preparations. The slides were air dried overnight, then for each condition of treatment, the number of breaks and gaps was observed on Giemsa-stained metaphases. For each time point, at least 50 chromosome metaphases were examined by two independent investigators, and chromosomal damage was scored at 100× magnification with an Olympus fluorescence microscope.

***In situ* PLA assay**

The *in situ* proximity-ligation assay (PLA; Olink, Bioscience) was performed according to the manufacturer's instructions and as previously described [4]. Briefly, antibody staining was carried out according to the standard immunofluorescence procedure. The primary antibodies used were: mouse-monoclonal anti-RNA-DNA hybrids, S9.6 (Kerafast), rabbit-polyclonal anti-RAD51 (Abcam) and mouse-monoclonal anti-IdU/BrdU (Beckton-Dickinson). The negative control consisted of using only one primary antibody. Samples were incubated with secondary antibodies conjugated with PLA probes MINUS and PLUS: the PLA probe anti-mouse PLUS and anti-rabbit MINUS (OLINK Bioscience). The incubation with all antibodies was accomplished in a moist chamber for 1 h at 37°C. Next, the PLA probes MINUS and PLUS were ligated using two connecting oligonucleotides to produce a template for rolling-cycle amplification. After amplification, the products were hybridized with red fluorescence-labelled oligonucleotide. Samples were mounted in ProLong Gold antifade reagent with DAPI (blue). Images were acquired randomly using Eclipse 80i Nikon Fluorescence Microscope, equipped with a VideoConfocal (ViCo) system.

Supplementary legends to figures

Figure S1. Down-regulation of WRN using siRNA performed in MRC5SV and shWRNIP1 cells and verified by WB. The membrane was probed with the indicated antibodies.

Figure S2. WRNIP1 is recruited to chromatin in a cell type-independent manner. WB detection of chromatin binding of WRNIP1 in total extracts of WRN-wt and WRN-kd cells untreated (-) or treated with Aph, as indicated. In WRN-kd cells, down-regulation of the WRN protein was verified using an anti-WRN antibody (Input). Chromatin fractionation was performed under low (100 mM) or high (300 mM) salt concentrations. The membrane was probed with the indicated antibodies. The normalized ratio of the chromatin-bound WRNIP1/LAMIN B1 signal (chromatin) is reported for both extraction conditions.

Figure S3. Depletion of WRNIP1 using RNAi performed in WSWRN and WS cells and verified by WB.

Figure S4. Loss of WRN helicase activity does not affect ATM activation. (**A**) WB analysis of chromatin binding of WRNIP1 in WS cells, wild-type (WSWRN*)* or WS cells expressing a mutant form of WRN affecting helicase function (WRN^{K577M}) treated or not with Aph. Chromatin fractionation of cell lysates was performed under low (100 mM) or high (300 mM) salt concentrations as reported in Fig. 2A. The membrane was probed with the indicated antibodies. The normalized ratio of the chromatin-bound WRNIP1 in the high salt concentrations cell extract/the chromatin bound WRNIP1 in the low salt concentrations extract is reported. (**B**) WB analysis of chromatin binding of WRNIP1 in WRN^{K577M} cells exposed or not to Aph, and/or to 300 nM of CHK1 inhibitor, UCN-01, for the last 6 h. Chromatin fractionation of cell lysates was performed under high (300 mM) salt concentrations. The membrane was probed as above. The ratio of the chromatin-bound WRNIP1 normalized to the untreated is reported below each line. (**C**) WB analysis of chromatin binding of WRNIP1 in WSWRN cells exposed or not to Aph, and/or to 300 nM UCN-01. Chromatin fractionation of cell lysates was performed as above. The ratio of the chromatin-bound WRNIP1 normalized to the untreated is reported below each line.

Figure S5. Down-regulation of Claspin and TopBP1 performed in WSWRN cells using specific siRNAs and verified by WB. The membrane was probed with the indicated antibodies.

Figure S6. TopBP1 depletion in WSWRN cells triggers checkpoint activation upon prolonged exposure to Aph. WB analysis of CHK1 phosphorylation in WSWRN cells transfected with GFP or WRNIP1 siRNA and treated or not with Aph for 8 and 24 h. After transfection, the presence of activated, i.e. phosphorylated, CHK1 was assessed using S345 phospho-specific antibody (pS345). The normalized ratio of the phosphorylated CHK1/total CHK1 is given.

Figure S7. Depletion of WRNIP1 using siRNA performed in WS cells and verified by WB. The membrane was probed with the indicated antibodies.

Figure S8. Depletion of WRNIP1 and TopBP1 using specific siRNAs performed in WSWRN cells and verified by WB. The membrane was probed with the indicated antibodies.

Figure S9. Representative images for parental ssDNA exposure in WSWRN and WS cells upon exposure to Aph for different times. DNA was counterstained with DAPI (blue).

Figure S10. WB analysis of level of FLAG-tag in WS cells after transfection with empty vector or FLAG-tagged $CHK1^{317/345D}$ and treated with Aph. The membrane was probed with the indicated antibodies.

Figure S11. Representative images of parental ssDNA exposure in WSWRN and WS cells after transfection with a plasmid expressing GFP-tagged RNaseH1 and treatment with Aph.

Figure S12. XPG depletion leads to increased exposure of ssDNA in WS cells. Evaluation of ssDNA accumulation at parental-strand by immunofluorescence analysis in WS cells transfected with control siRNAs (siCtrl) or siRNAs directed against XPG (siXPG). After 48 h, cells were treated as reported in the experimental scheme. After treatment, cells were fixed and stained with an anti-IdU antibody without denaturing the DNA to specifically detect parental ssDNA. Expression levels of XPG were determined by immunoblotting with anti-XPG antibody.

Figure S13. TopBP1 depletion leads to transcription-dependent exposure of ssDNA in WSWRN cells. Representative images for Figure 6D are given. DNA was counterstained with DAPI (blue).

Figure S14. Uncropped blots for Figures 1A and S1.

Figure S15. Uncropped blots for Figures 2B, 2D, 2E and S3.

Figure S16. Uncropped blots for Figures 3A, 3C and S5.

Figure S17. Uncropped blots for Figures S7 and S8.

Figure S18. Uncropped blots for Figures 5A, 5B, 5C and 5D.

Figure S19. Uncropped blots for Figure 6E.

Figure S20. Uncropped blots for Suppl. Figure 2.

Figure S21. Uncropped blots for Suppl. Figures 4A, 4B and 4C.

Figure S22. Uncropped blots for Suppl. Figures 6, 10 and 12.

REFERENCES

1. Basile, G., Leuzzi, G., Pichierri, P. & Franchitto, A. Checkpoint-dependent and independent roles of the Werner syndrome protein in preserving genome integrity in response to mild replication stress. *Nucleic Acids Res.* **42,** 12628–12639 (2014).
2. Gatei, M. *et al.* Ataxia-telangiectasia-mutated (ATM) and NBS1-dependent Phosphorylation of Chk1 on Ser-317 in Response to Ionizing Radiation. *J. Biol. Chem.* **278,** 14806–14811 (2003).
3. Cerritelli, S. M. *et al.* Failure to produce mitochondrial DNA results in embryonic lethality in Rnaseh1 null mice. *Mol. Cell* **11,** 807–815 (2003).
4. Leuzzi, G., Marabitti, V., Pichierri, P. & Franchitto, A. WRNIP 1 protects stalled forks from degradation and promotes fork restart after replication stress. *EMBO J.* **35,** 1–15 (2016).
5. Pichierri, P., Franchitto, A., Mosesso, P., Palitti, F. & Molecolare, C. Werner ' s Syndrome Protein Is Required for Correct Recovery after Replication Arrest and DNA Damage Induced in S-Phase of Cell Cycle. *Mol. Biol. Cell* **12,** 2412–2421 (2001).
6. Pirzio, L. M., Pichierri, P., Bignami, M. & Franchitto, A. Werner syndrome helicase activity

is essential in maintaining fragile site stability. *J. Cell Biol.* **180,** 305–314 (2008).
