# Supplementary material for: Checkpoint Defects Elicit a WRNIP1-Mediated Response to Counteract R-Loop-Associated Genomic Instability
Source: Cancers (Basel). 2020 Feb 7;12(2):389. doi: 10.3390/cancers12020389 (PMC7072626; doi:10.3390/cancers12020389)
Supplement: Supplementary file 1 [file cancers-12-00389-s001.zip › Cancers supplementary figures.pdf]

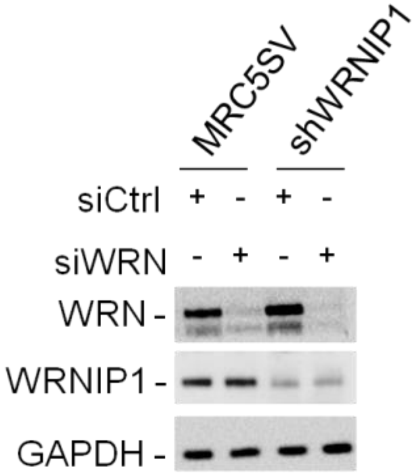

Suppl. Figure 1

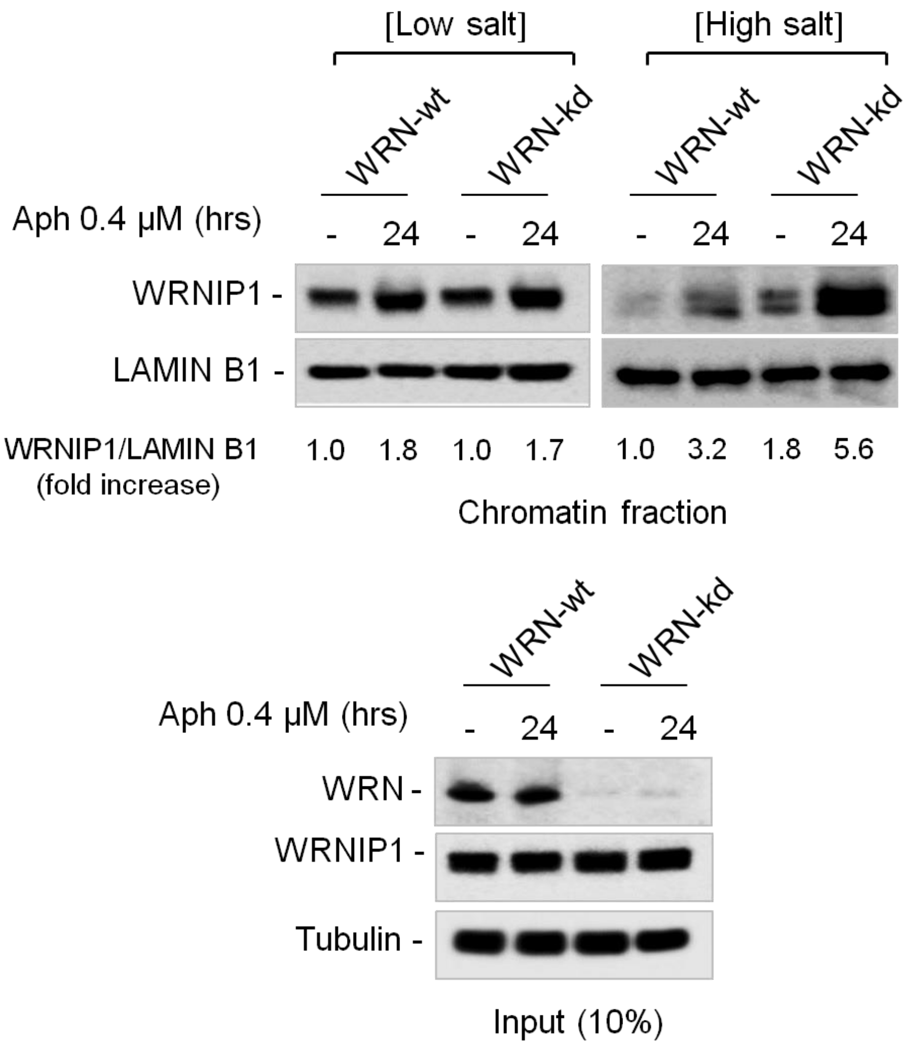

Suppl. Figure 2

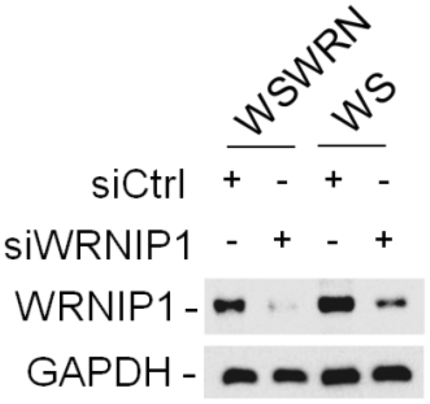

Suppl. Figure 3

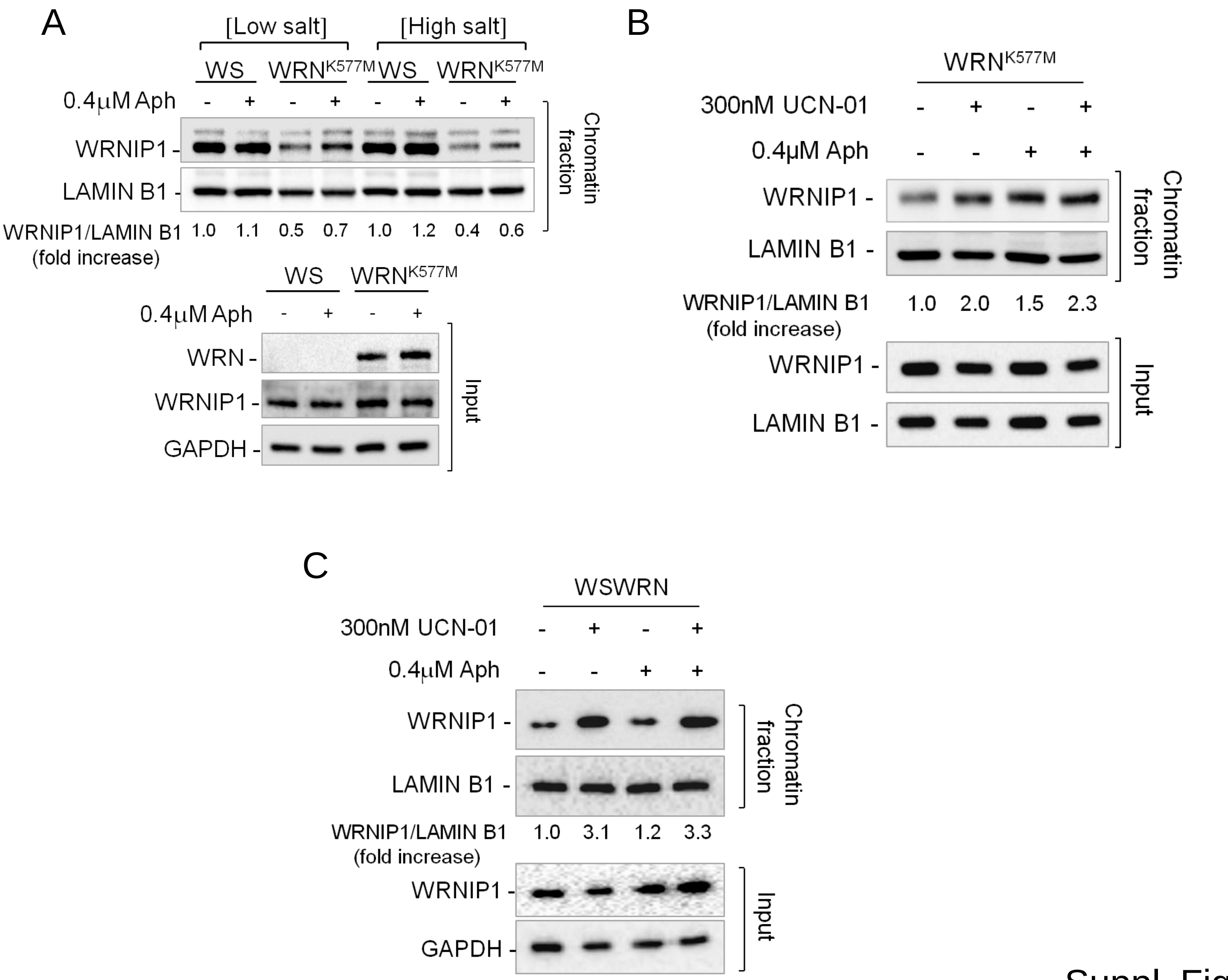

Suppl. Figure 4

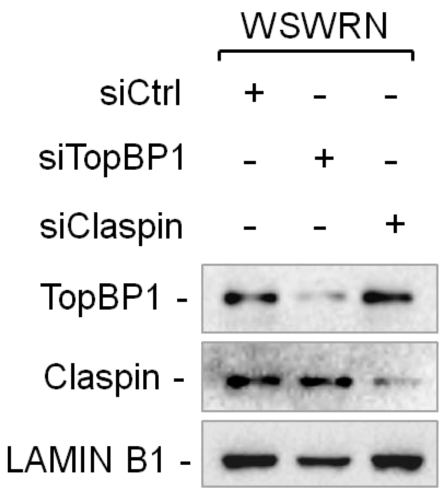

Suppl. Figure 5

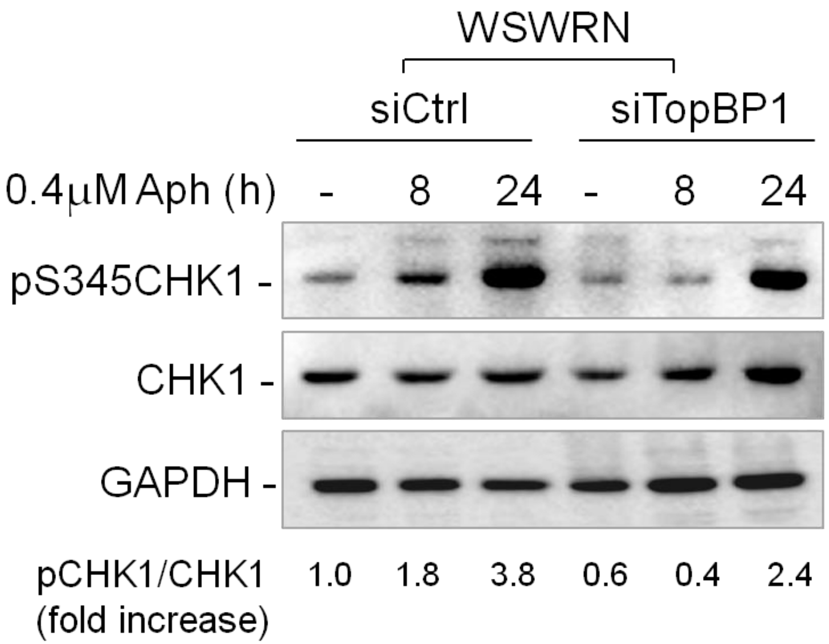

Suppl. Figure 6

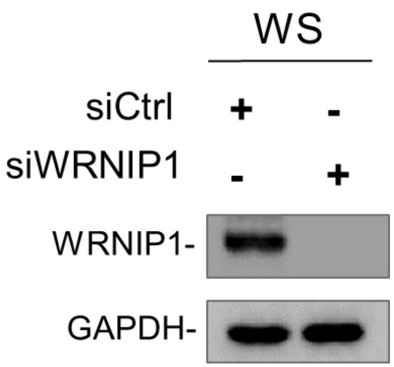

Suppl. Figure 7

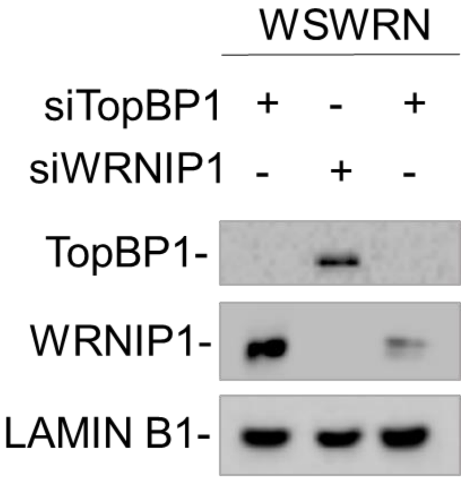

Suppl. Figure 8

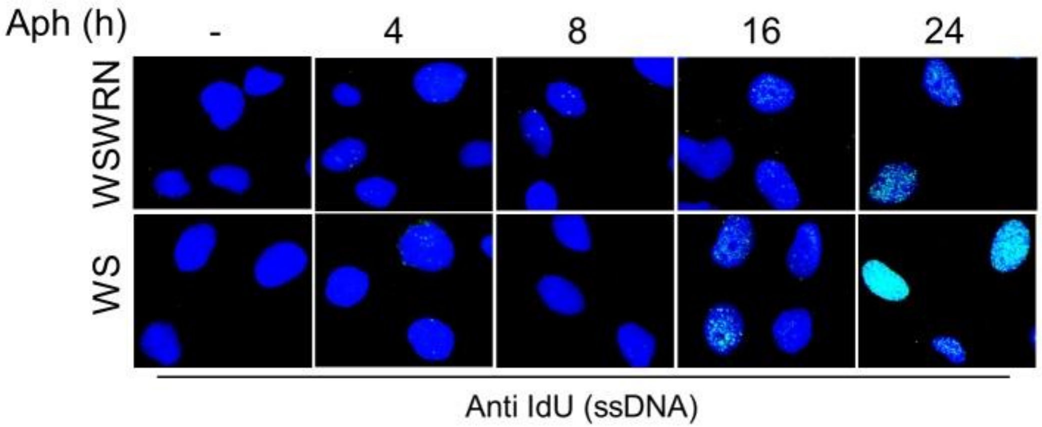

Suppl. Figure 9

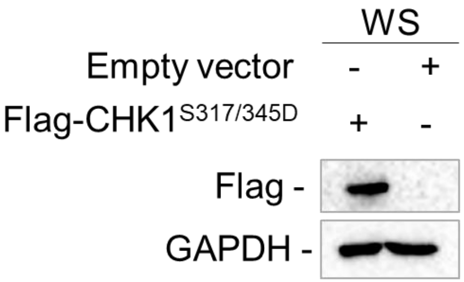

Suppl. Figure 10

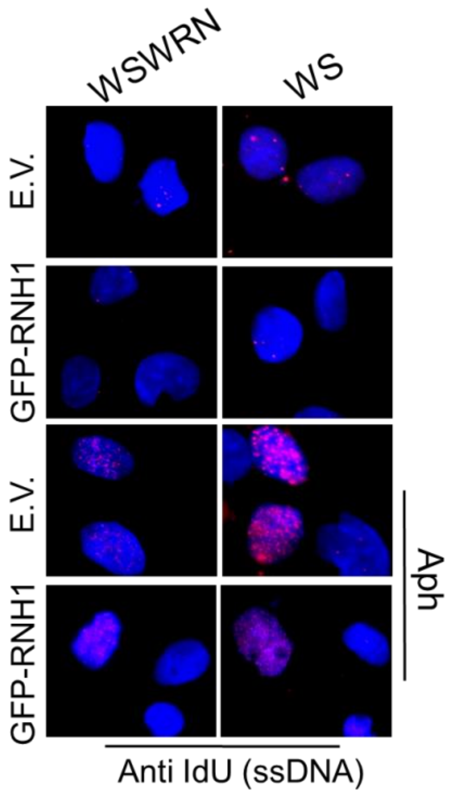

Suppl. Figure 11

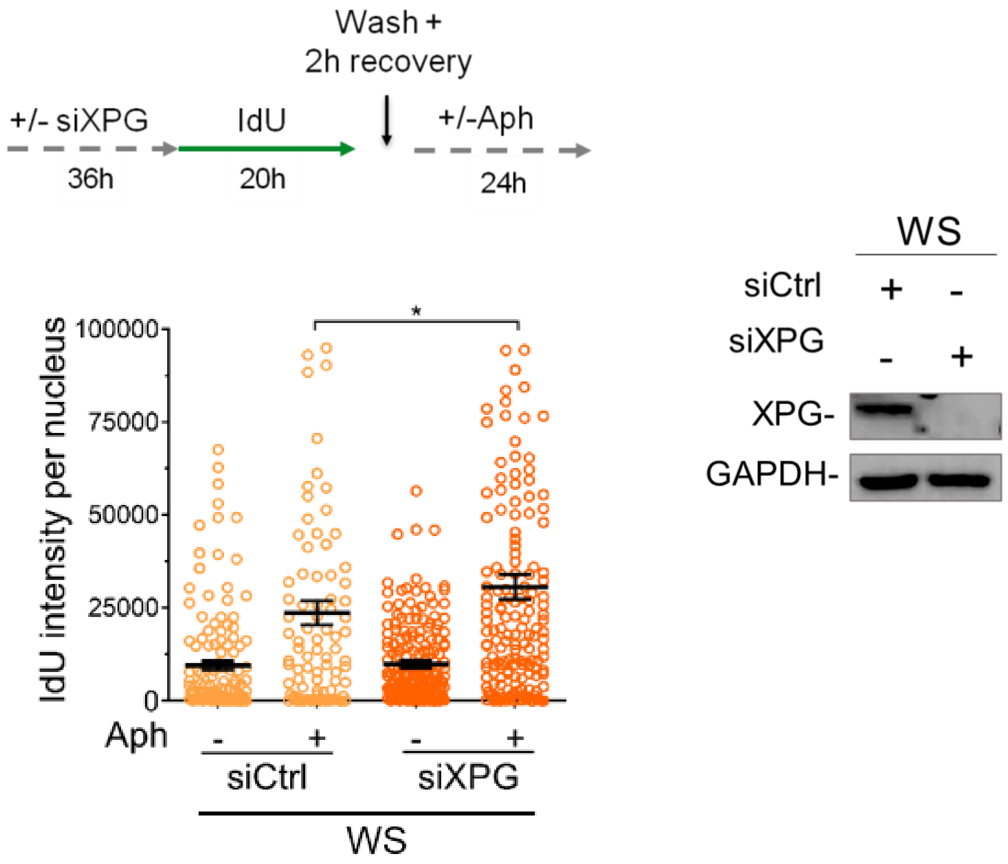

Suppl. Figure 12

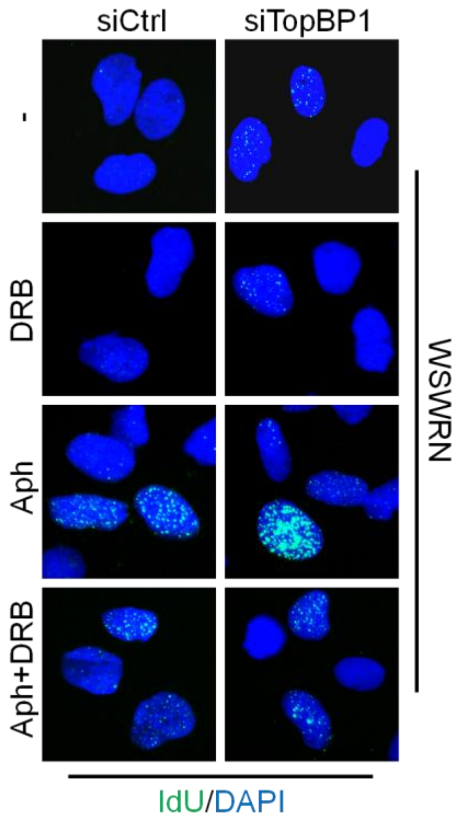

Suppl. Figure 13

Figure 1

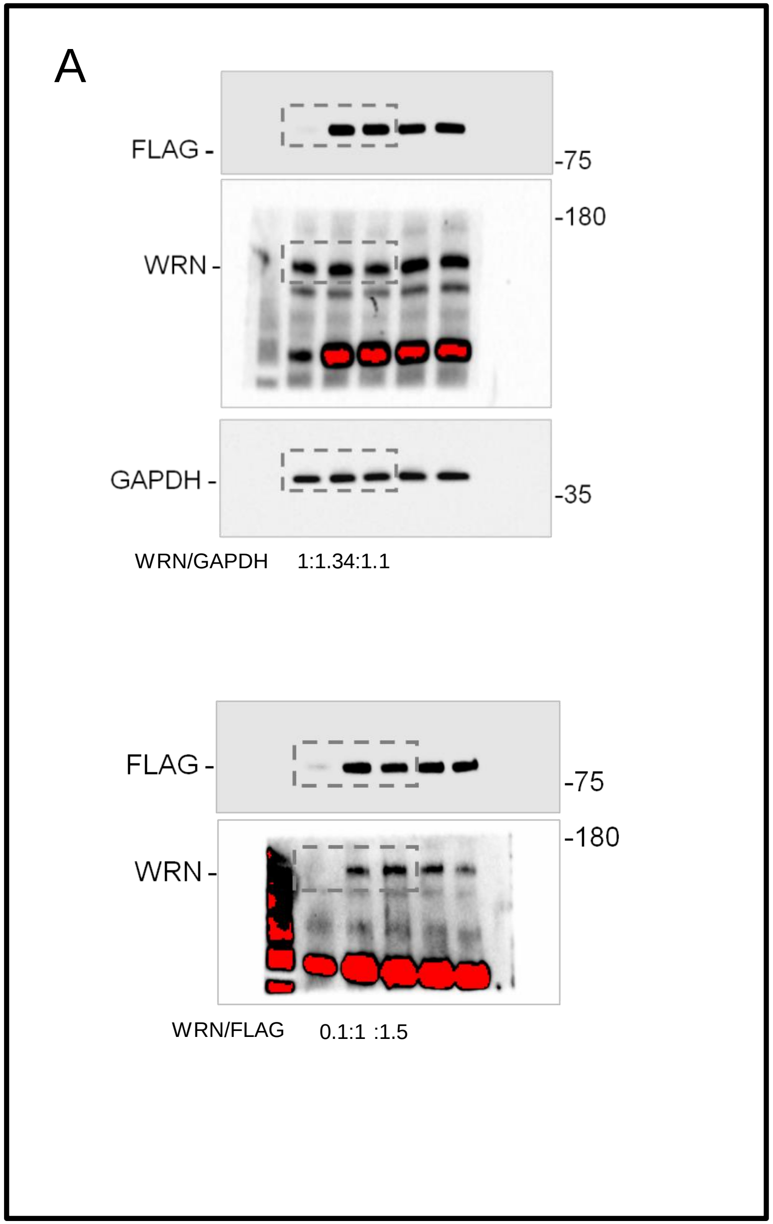

Suppl. Figure 1

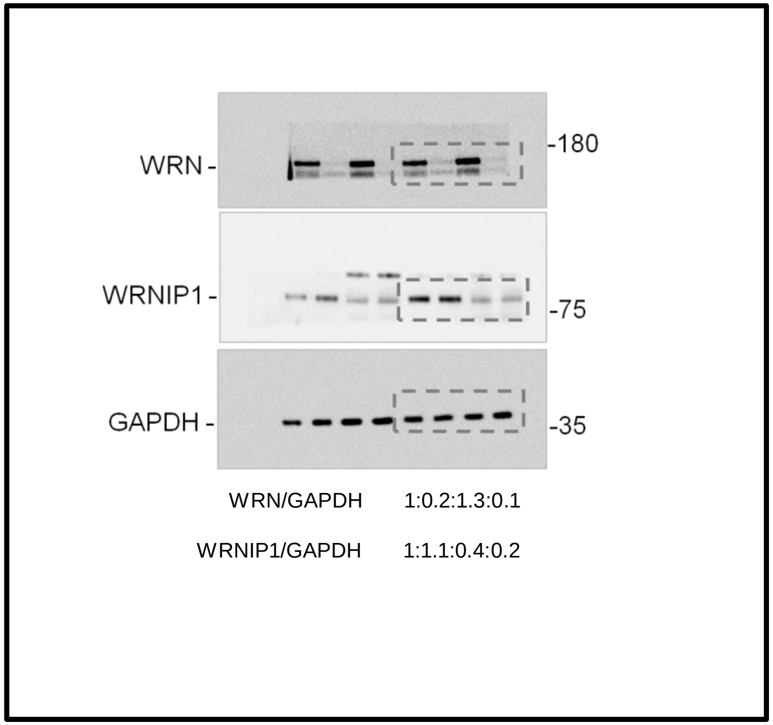

Supplementary Fig. 14

Figure 2

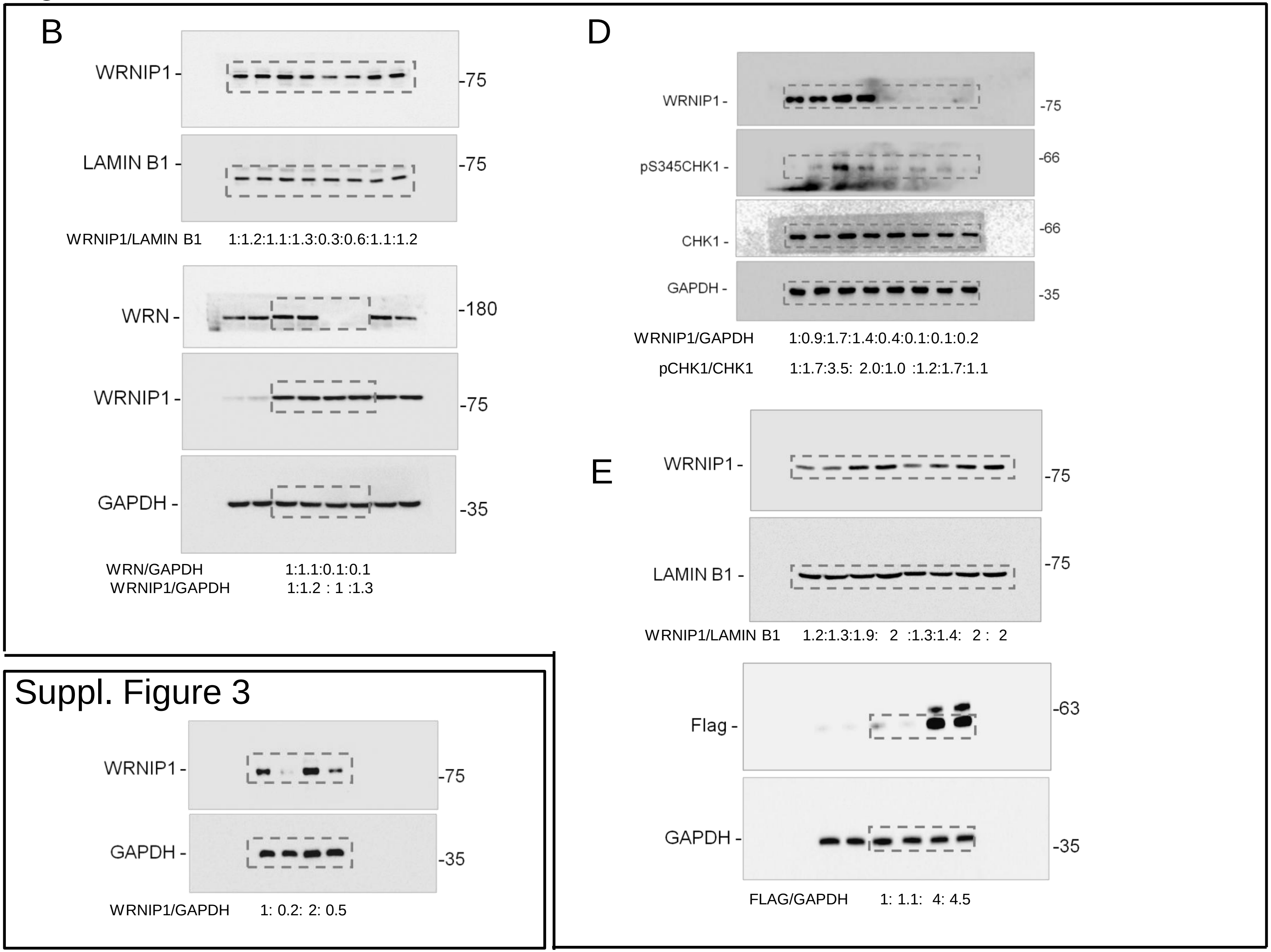

Supplementary Fig. 15

Figure 3

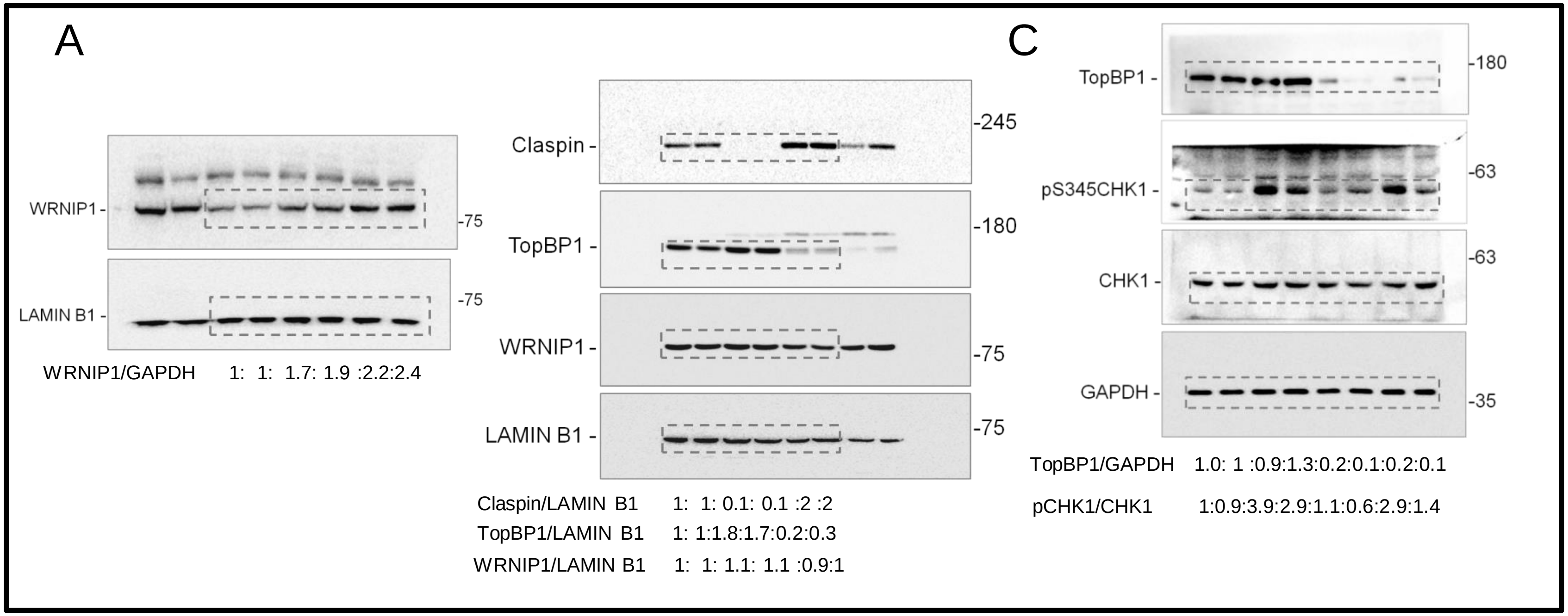

Suppl. Figure 5

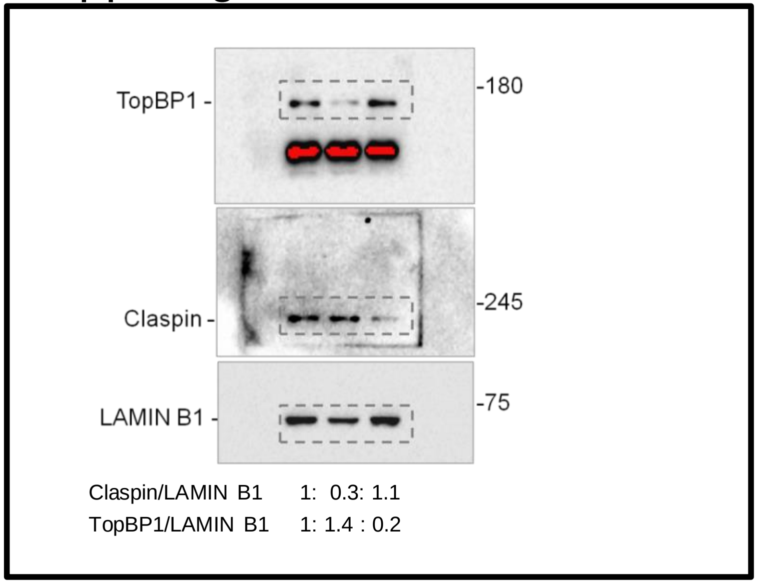

Supplementary Fig. 16

Suppl. Figure 7

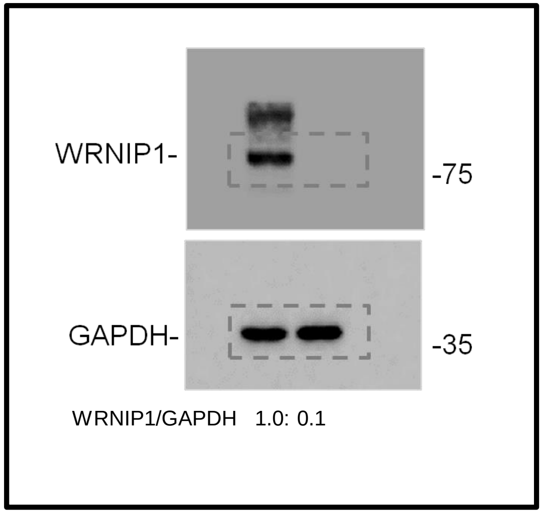

Suppl. Figure 8

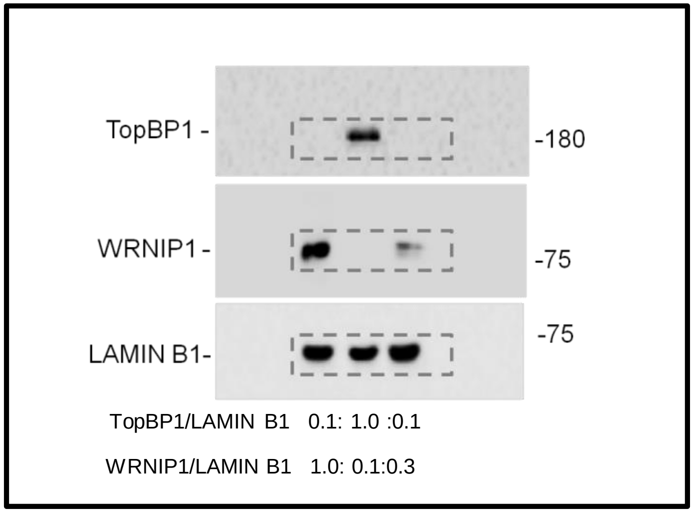

Figure 5

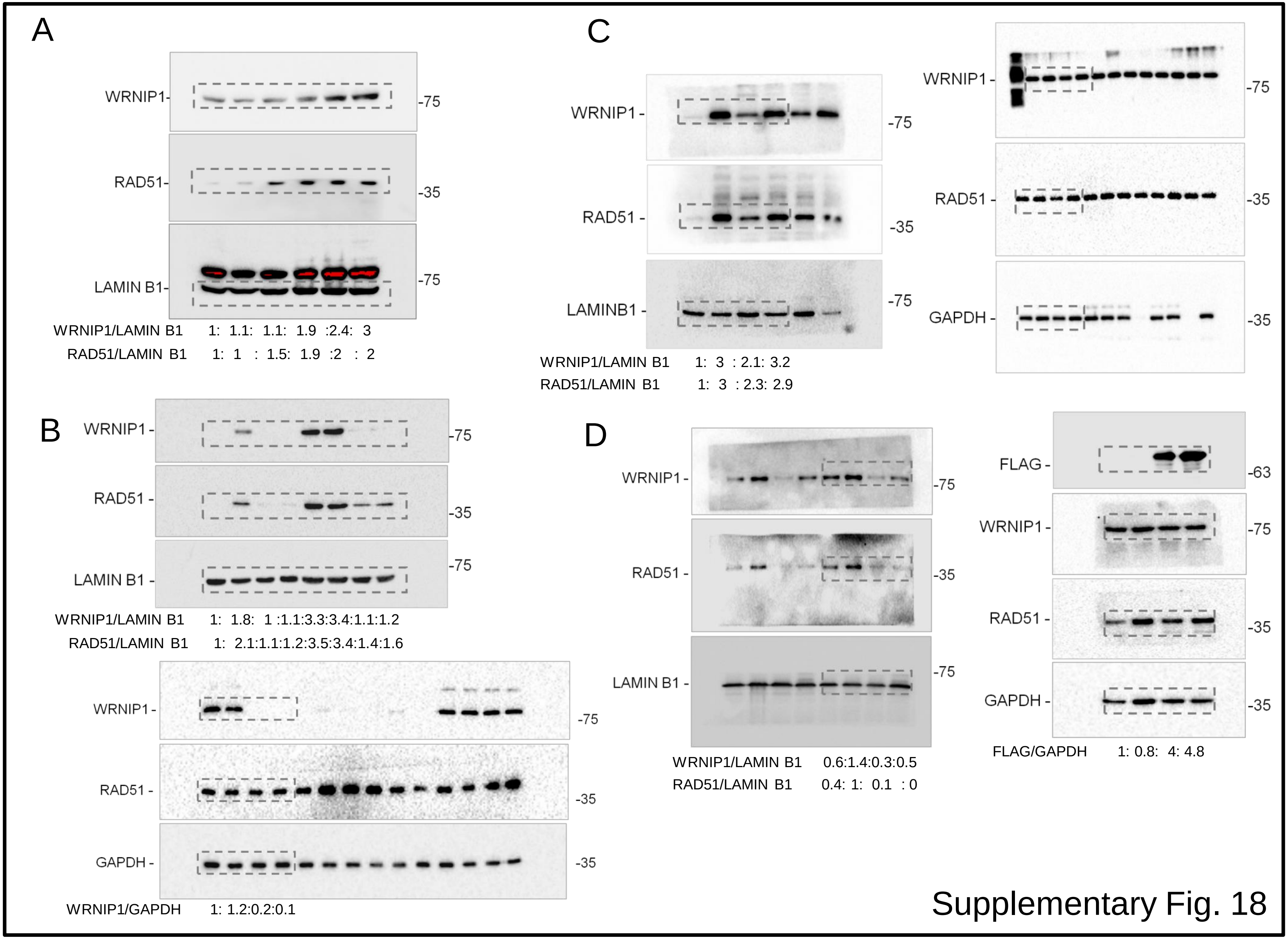

Supplementary Fig. 18

Figure 6

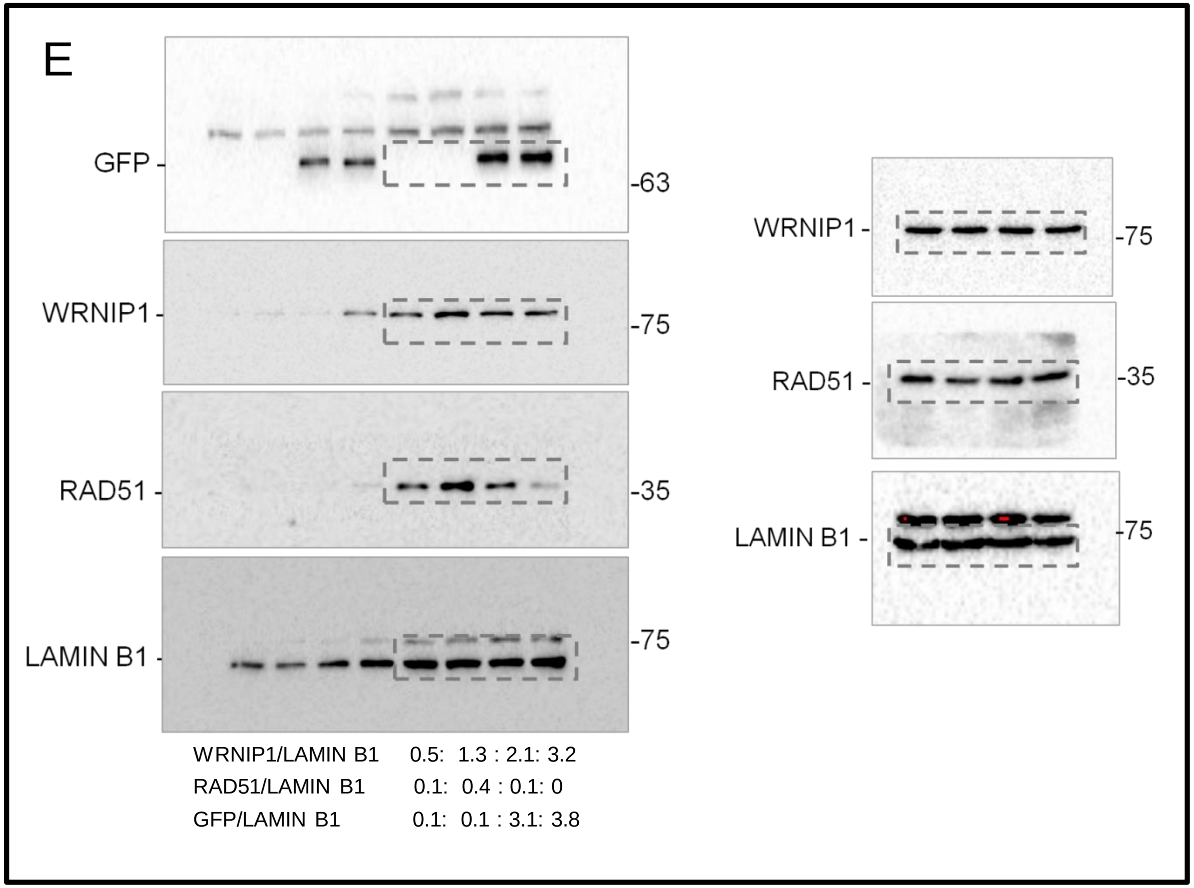

E
GFP
-63
WRNIP1
-75
RAD51
-35
LAMIN B1
-75
WRNIP1/LAMIN B1 0.5: 1.3 : 2.1: 3.2
RAD51/LAMIN B1 0.1: 0.4 : 0.1: 0
GFP/LAMIN B1 0.1: 0.1 : 3.1: 3.8
WRNIP1
-75
RAD51
-35
LAMIN B1
-75

Suppl. Figure 2

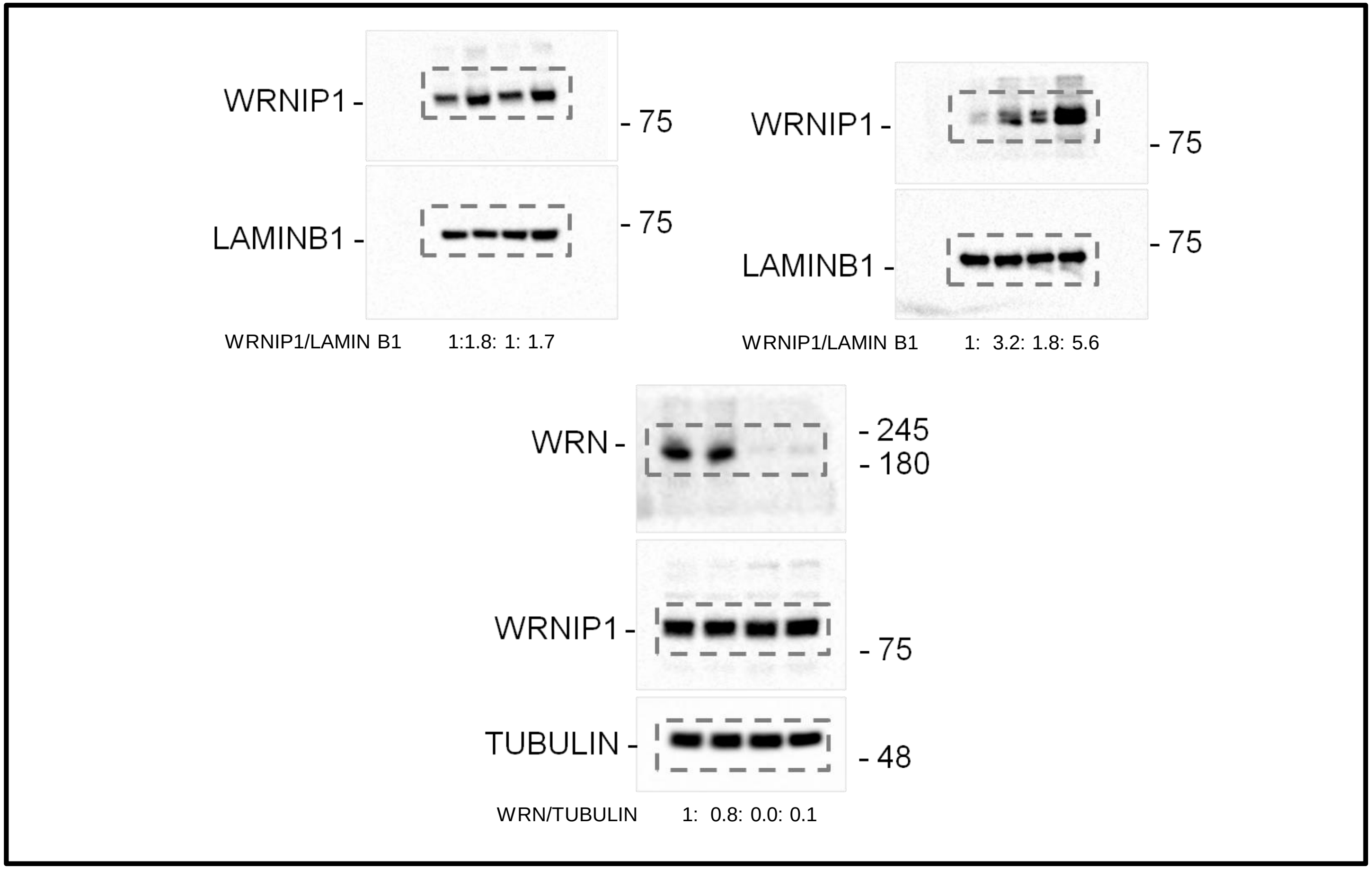

Suppl. Figure 4

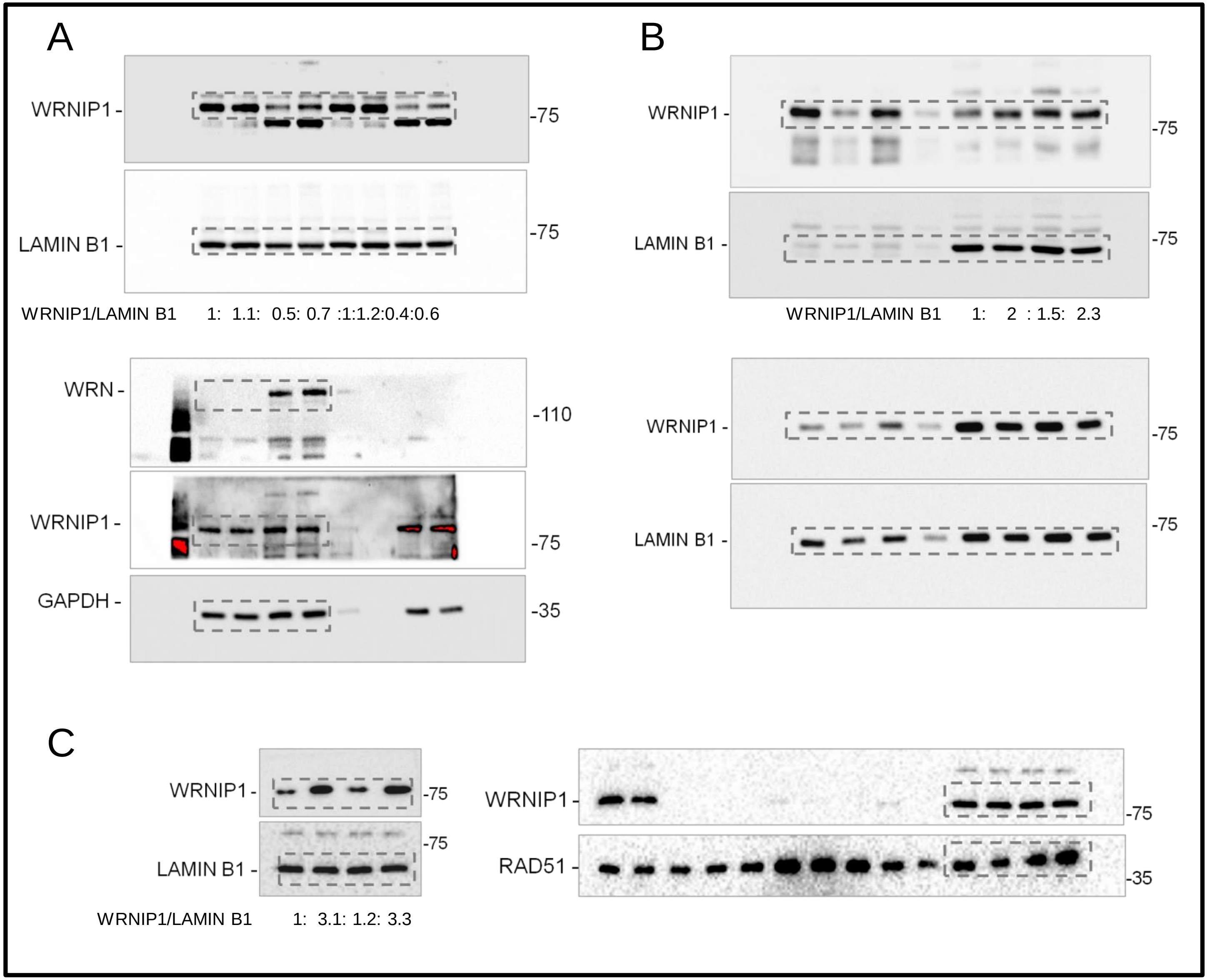

Supplementary Fig. 21

Suppl. Figure 6

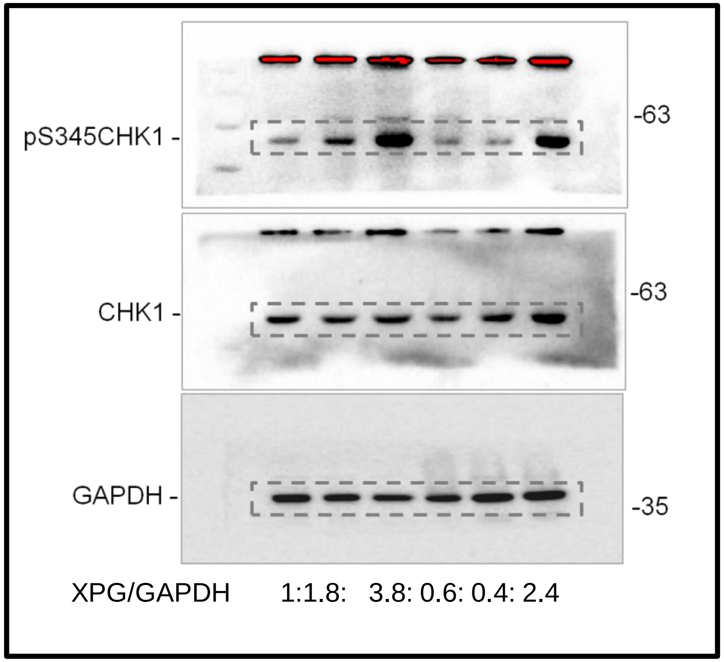

Suppl. Figure 10

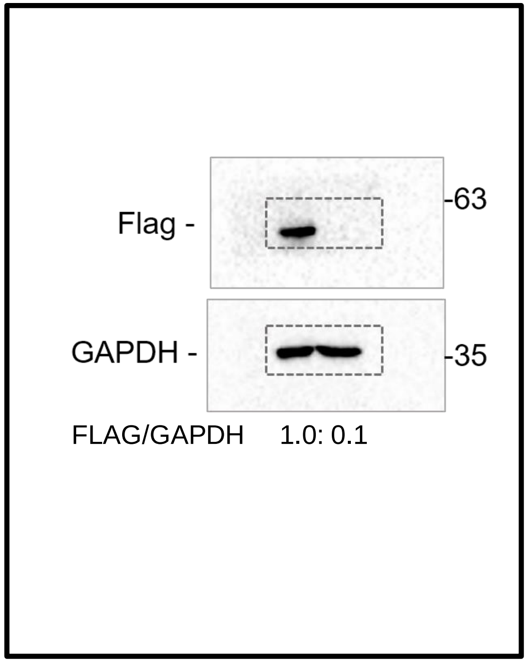

Suppl. Figure 12

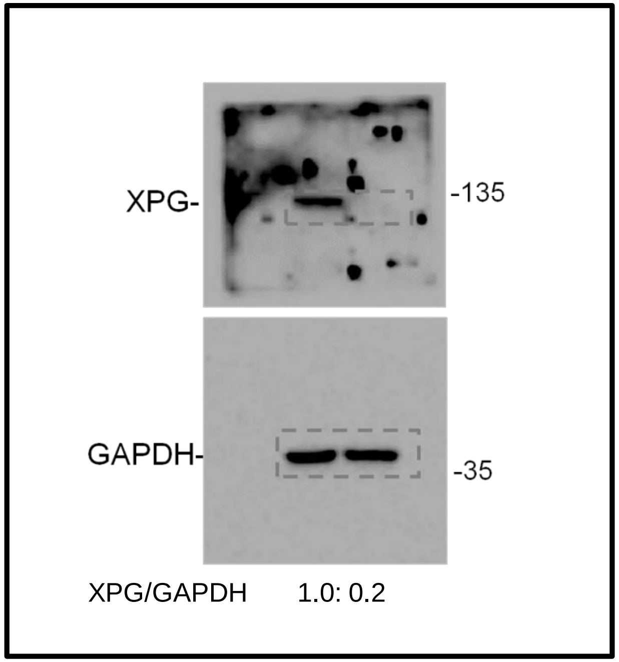

Supplementary Fig. 22
